# Announcement: Community Preventive Services Task Force Recommendation for Interventions Including Activity Monitors to Increase Physical Activity in Adults with Overweight or Obesity

**DOI:** 10.15585/mmwr.mm6643a8

**Published:** 2017-11-03

The Community Preventive Services Task Force (CPSTF) recommends interventions that include activity monitors to increase physical activity in adults with overweight or obesity. “Physical Activity: Interventions Including Activity Monitors for Adults with Overweight or Obesity” is available at https://www.thecommunityguide.org/findings/physical-activity-interventions-including-activity-monitors-adults-overweight-obesity.

Established in 1996 by the U.S. Department of Health and Human Services, the CPSTF is an independent, nonfederal panel of public health and prevention experts whose members are appointed by the director of CDC. The CPSTF provides information for a wide range of persons who make decisions about programs, services, and other interventions to improve population health. Although CDC provides administrative, scientific, and technical support for the CPSTF, the recommendations developed are those of the task force and do not undergo review or approval by CDC.

